# Measuring a motivational interviewing practice orientation in criminal justice practitioners: initial validation of the response style screening questionnaire

**DOI:** 10.3389/fpsyg.2023.1308086

**Published:** 2023-12-06

**Authors:** Raymond Chip Tafrate, Damon Mitchell, Stephen M. Cox, Tom Hogan

**Affiliations:** ^1^Department of Criminology and Criminal Justice, Central Connecticut State University, New Britain, CT, United States; ^2^Institute for the Study of Crime and Justice, Central Connecticut State University, New Britain, CT, United States

**Keywords:** motivational interviewing, community corrections, probation, probation officer skill assessment, response style screening questionnaire

## Abstract

**Introduction:**

The use of collaborative communication techniques by criminal justice practitioners has been identified as a component of core correctional practices (CCPs). Criminal justice agencies and programs are investing in motivational interviewing (MI) training for their staff with varying intensity, follow-up coaching, and expectations for integration into practice. The present article describes the development and initial validation of the Response Style Screening Questionnaire (RSSQ), a practitioner completed tool assessing an MI-consistent practice orientation. Over three studies, we examined the factor structure, reliability, and correlations between the scale and self-reported and behavioral validity indicators.

**Method:**

Study 1 examined the factor structure of the RSSQ with a sample of 825 criminal justice practitioners. In Study 2, data from 350 probation officers completing the RSSQ alongside measures of work-orientation and attitudes was used to conduct a confirmatory factor analysis and an initial assessment of its construct validity. In Study 3, correlations between the RSSQ and audio recorded office visits of 33 probation officers coded for MI and relationship building skills examined the scale’s criterion validity.

**Results:**

In Study 1, an exploratory factor analysis with an oblique rotation yielded 18 items on four factors. In Study 2, four and five factor models were tested, with the 4-factor model of Study 1 yielding the best fit. Two of the 4 factor-derived subscales reflect styles inconsistent with MI: (1) Confrontational style, and (2) Sustain Talk style; while the remaining two reflect styles consistent with MI: (3) Eliciting style, and (4) Change Talk style. Confrontational style scores were correlated with a work-orientation reflecting probation as a law enforcement endeavor, while Eliciting and Change Talk scores were correlated with a behavior change and resource broker work-orientations. In Study 3, Confrontational and Sustain Talk style scores were negatively correlated with a variety of MI skills and CCPs displayed on audio recordings, while Change Talk style scores were positively correlated with use of such skills.

**Discussion:**

Overall, the findings suggest the RSSQ is a potentially useful new practitioner self-report tool for assessing an MI practice orientation.

## Introduction

Across a variety of criminal justice settings, forensic practitioners face a common set of challenges in their work with justice-involved individuals. Foremost, is engaging a population of clients who are generally mandated or coerced into services (e.g., treatment, case management, intervention, etc.) they do not want and/or do not believe they need. For example, in intimate partner violence intervention programs, client motivation and program adherence are common roadblocks to treatment effectiveness (Eckhardt et al., 2013; Lila et al., 2018). Second, criminal justice policy goals of “improving public safety” and “reducing recidivism” place expectations on forensic practitioners to positively influence the behavioral trajectories of their clients, beyond the traditional emphasis on surveillance, drug testing, and imposing “accountability.” Accomplishing these tasks requires addressing clients’ criminogenic needs (Bonta and Andrews, 2023), often necessitating a focus on sensitive and difficult-to-discuss topics such as family violence, sex offending, and problematic substance use, among others. To that end, motivational interviewing (MI) has established itself as a foundational skill set that is currently integrated into forensic settings (Stinson and Clark, 2017; Tafrate et al., 2019). MI can be valuable in establishing rapport, guiding conversations in productive directions, and allowing practitioners to explore and heighten clients’ awareness of the connections between lifestyle choices and subsequent losses (e.g., damaged relationships, ruined career paths, financial problems, and incarcerations), fostering motivation around changing criminogenic needs and other risky behaviors. Skilled practitioners can also integrate MI into the assessment process by connecting assessment to clients’ real-life challenges and valued goals, as well as providing meaningful feedback on the results (Aschieri et al., 2023).

An early review by McMurran (2009) indicated that MI has the potential to positively influence how clients respond to mandated services; enhancing retention, increasing motivation to change destructive behaviors, and reducing reoffending. Recent reviews exploring MI and substance use among justice-involved individuals have produced mixed conclusions; one review found insignificant results (Pederson et al., 2021), while a second suggested MI interventions had a preventative effect on intoxicated driving (Razaghizad et al., 2021). Several reviews of programs directed at perpetrators of intimate partner violence have converged around the conclusion that incorporating MI had positive effects on program adherence, attrition, motivation, and equivocal results regarding recidivism (Santirso et al., 2020; Pinto e Silva et al., 2023), suggesting optimism with this challenging client group.

As community supervision continues to evolve, practitioners struggle with acquiring more complex skills—as well as shifting roles, underlying job motivations, and responsibilities (Bourgon et al., 2012; Lovins et al., 2018). Agencies and programs invest heavily in training their staff to make sure they are knowledgeable and competent in bringing empirically supported practices into everyday interactions. To assess the results of training in MI and other communication and intervention skills, agencies and programs often implement some form of *quality assurance* processes. With respect to MI skills, administrators may wish to (a) evaluate baseline competence and orientation (at various levels such as agency/program, office, and individual), (b) assess the impact of training initiatives on knowledge and skills, (c) improve MI proficiency after training through feedback and coaching, and (d) identify practitioners who may be causing harm and require additional oversight and training (Moyers and Miller, 2013).

## Measuring forensic practitioners’ MI skills: a review of assessment strategies

An ample literature-base exists around the assessment of MI fidelity and skill, and numerous tools have emerged with varying degrees of psychometric evidence. For a comprehensive overview, readers are referred systematic reviews of MI-relevant instruments (Dobber et al., 2015; Hurlocker et al., 2020), several meta-analyses on the effects of MI training (Schwalbe et al., 2014; Madson et al., 2019), and a narrative description of developments in assessing the quality of MI conversations (Miller and Rollnick, 2023, pp. 285–297). For the purposes of the present study, we restrict our review to the strengths and weaknesses of various MI assessment strategies employed in criminal justice settings, highlighting some of the more established tools.

### Practitioner observation tools

Listening to live practice is one of the best ways to evaluate MI skills. Since practitioner observation tools are complex, recorded work samples (e.g., audio) are typically coded by a trained observer guided by behavioral descriptions of global dimensions (e.g., empathy, partnership, cultivating change talk, softening sustain talk), frequency of MI-consistent and non-consistent practitioner behaviors (e.g., questions, simple reflections, affirmations, confrontational statements, persuasion), and, in some cases, client verbalizations (i.e., change talk and sustain talk). Global dimensions are often rated on a 5-point Likert scale with a rating of one representing the lowest level of skill and five being the highest, while practitioner behaviors are coded, counted, and combined to calculate summary scores that can be compared to competency thresholds.

The most comprehensive tool is the Motivational Interviewing Skills Code (MISC; Houck et al., 2010) which measures both practitioner and client responses. In a recent review, MISC reliability estimates were found to be variable (i.e., excellent ratings of global dimensions and poor-to-good for practitioner behavior counts), with some encouraging findings for predictive validity, and little research into its construct validity (Hurlocker et al., 2020). Although the MISC provides the most complete picture of practitioner-client interactions, it is a labor-intensive instrument as raters must listen to a work sample three times to code a practitioner-client interaction.

The most common tool in both training and efficacy studies is the Motivational Interviewing Treatment Integrity (MITI; Moyers et al., 2014, 2016), which is designed to be less time-intensive because it is focused solely on practitioner behaviors. Reliability estimates on the early version of the MITI were variable, however the most recent version has demonstrated good-to-excellent reliability for global dimensions and practitioner behavior counts, and strong criterion validity (Hurlocker et al., 2020).

Relatively few studies have used MISC or MITI in criminal justice settings. Rodriguez et al. (2018) examined MI session recordings of 40 substance-using probationers with the MISC and found moderate to good levels of inter-rater reliability. In addition, practitioner MI-adherent skills were associated with greater client change talk, while client sustain talk predicted continued substance use. In a similar study of 40 substance-using probationers, Spohr et al. (2016) examined recordings using the MITI and found fair-to-excellent levels of reliability between coders. Moreover, practitioner MI adherent relational skills (e.g., empathic, collaborative, emphasizing client autonomy) predicted client-initiated treatment at follow-up, but were unrelated to subsequent substance use. Owens et al. (2017) examined the global dimensions of the MITI in a sample of 22 prison inmates with substance use histories who received a brief MI intervention prior to release. The coding scheme demonstrated strong inter-rater reliability and initial evidence of predictive validity in that *cultivating change talk* was associated with post-intervention ratings of motivation to decrease drug use, but not with other self-reported outcomes such as alcohol use or abstinence.

It should be noted that in all three studies mentioned above, MI sessions were delivered by practitioners (e.g., graduate students; counselors) who were trained/supervised by experienced MI researchers in the context of a clinical trial, and not delivered by actual probation or correctional staff. While these studies suggest optimism regarding use of the MISC and MITI with forensic clientele, there is a dearth of information on real-world implementation of these tools in criminal justice settings.^1^

Although less well-known, several other practitioner rating tools have relevance for criminal justice professionals. Lane et al. (2005) developed the Behavior Change Counseling Index (BECCI) to assess MI proficiency in healthcare settings. This tool contains a checklist of 11 items (e.g., practitioner encourages client to talk about change, uses empathic listening, conveys respect for client choice) that are measured on a 5-point Likert-type scale ranging from 0 (not at all) to 4 (to a great extent). Across studies the BECCI has demonstrated poor-to-good internal consistency, adequate-to-excellent inter-rater reliability, and no estimates of validity (Hurlocker et al., 2020). A parallel tool designed for probation settings is the BECC-CJ (Motivational Interviewing Network of Trainers, 2011). Although this tool shows promise as a measure of MI practice for forensic practitioners, no studies have evaluated the BECCI-CJ.

A newer tool, validated with 14 youth justice program practitioners, is the Motivational Interviewing Evaluation Rubric (MIER; Báez et al., 2020). The MIER is a 15-item observation tool that measures the subcategories of MI *spirit*, *process,* and *skills* from brief recordings (20 min or less). In an initial validation study, subscale scores showed good internal consistency and excellent inter-rater reliability, moderate to substantial reliability on individual items, as well as good convergent validity with the MITI. The MIER appears promising as a concise alternative for assessing MI fidelity in forensic settings.

Although equivocal findings exist, practitioner observation tools such as the MISC and MITI have emerged with the greatest empirical support and are considered the gold standard in assessing MI practice (Hurlocker et al., 2020). These instruments also lend themselves to providing individualized feedback to practitioners, which is beneficial in sustaining MI skills following training events. However, in the context of criminal justice environments, implementing validated practitioner observation tools may be impractical because of time demands in terms of training raters (up to 40 h of training; Moyers et al., 2016) and coding recordings (approximately 85 to 120 min of coding time per recording; Moyers et al., 2005), as well as ethical challenges in obtaining actual work samples. Also, a missing element in the validation of practitioner rating tools is that they have not strongly linked practitioner skills to client outcomes (Madson et al., 2019).

Because of these practical challenges, criminal justice agencies/programs sometimes settle for using brief *observation worksheets* to obtain snapshots of actual practice. Observation worksheets typically employ frequency counts of MI skills (e.g., number of open questions, reflections, affirmations, and summaries), as well as practitioner statements that are MI-inconsistent (e.g., closed questions, unsolicited advice, confrontation, threatening), and sometimes other markers of MI practice (e.g., empathy, evoking change talk). Snapshots can be taken from real-time observations (e.g., a supervisor can be in the room coding), through role-plays of practitioner-client interactions, or brief recorded work samples (e.g., practitioners can select their best 10 min of MI). This approach can side-step obstacles related to obtaining recordings, provide a written record that can be reviewed, and pinpoint practitioner “next steps” for improvement. Disadvantages of basic observation sheets is that they will not capture the nuances of MI practice and are often based on brief snippets of conversations. Not much is known about the validity of these tools, and they are difficult to locate in the literature. However, because such worksheets are easy to implement, we believe they are widespread in criminal justice settings. For examples see Motivational Interviewing Network of Trainers (2020, pp. 36–38), Martino et al. (2006, pp. 127–128), and Tafrate et al. (2018, p. 41).

### Practitioner completed tools

Another assessment strategy is to evaluate practitioners’ responses to a standard set of stimuli portraying typical client scenarios. Several instruments have adopted this approach and measure MI skills in the absence of real-world work samples. The Video Assessment of Simulated Encounters (VASE-R; Rosengren et al., 2008) consists of prompts inserted into three videotaped vignettes of actors playing clients with substance misuse. On most items, practitioners are asked to generate written responses consistent with MI, and for three additional multiple-choice items (one per vignette) to identify—from a list—practitioner statements likely to enhance motivation. All responses are rated/scored on a three-point scale (MI-inconsistent; neutral; MI-consistent), with higher scores indicating greater MI proficiency. In a recent review, the VASE-R emerged with acceptable-to-excellent inter-rater reliability estimates for the full-scale and subscale scores (e.g., reflective listening, responding to resistance, summarizing, eliciting change talk, and developing discrepancy), unacceptable-to-adequate alpha coefficients, and strong concurrent validity (Hurlocker et al., 2020). The VASE-R can be administered in about 30 min to both individual and groups, and vignettes representing other problem areas have been created (Boom et al., 2020).

Designed as a measure of accurate empathy, one of the first tools was the Helpful Responses Questionnaire (HRQ; Miller et al., 1991). The HRQ is a six-item paper and pencil test that presents hypothetical client statements (e.g., *A 59-year-old unemployed teacher tells you: “My life just does not seem worth living any more. I’m a lousy father. I cannot get a job. Nothing good ever happens to me. Everything I try to do turns rotten. Sometimes I wonder whether it’s worth it.”*) to which practitioners provide a written response of no more than two sentences (i.e., “the next thing that you would say”). Answers are then coded on a 5-point Likert scale where one indicates responses contrary to reflective listening and five indicates responses that capture the deeper meaning behind what a client said. The HRQ has demonstrated adequate internal consistency and good-to-excellent inter-rater reliability (Hurlocker et al., 2020), as well as some evidence of convergent validity (Miller and Mount, 2001; Smith et al., 2018). HRQ case vignettes have been expanded to different scenarios (Forrester et al., 2008; Hartzler, 2015), including applications for forensic practitioners described below.

The Officer Responses Questionnaire (ORQ; Walters et al., 2008) is an adaptation of the HRQ designed for use in community corrections. In completing the ORQ, practitioners provide a written response to five client statements that reflect typical probationer/parolee verbalizations (e.g., *A 24-year-old woman tells you: “I’ve been looking for work, but it’s impossible for someone on probation to find a good job”*). Scoring is similar to the HRQ with slight modifications (e.g., 1 = ordering, disagreeing, advice without permission; 2 = closed question, affirmation, offer to help; 3 = open question; 4 = simple reflection; 5 = deeper meaning reflection). In the initial validation study, good to excellent estimates of inter-rater reliability were reported, and probation officers (*n* = 80) showed significant improvement across all five items following a two-day MI training (Walters et al., 2008).

Another derivative of the HRQ is the Workers Responses Questionnaire (WRQ; Hohman et al., 2009). In this adaptation, practitioners provide a written response to five client statements consistent with what might be verbalized by justice-involved youth. Items are once again scored on 5-point Likert scale, with higher scores indicating greater consistency with MI principles. In a large implementation project with 576 corrections employees (a mix of counselors, security, and educational staff) who were mandated to attend a three-day MI training, WRQ scores indicated significant pre-to-post training improvements (Hohman et al., 2009).

Additional studies have used the VASE-R, ORQ, and WRQ in criminal justice settings. Exploring the effects of advanced MI training on 87 juvenile justice practitioners (a mix of probation, health care, and supervisors), Hartzler and Espinosa (2010) reported significant pre-to-post training increases in MI skillfulness as measured by the VASE-R. In an implementation project, Doran et al. (2011) used the VASE-R and WRQ to assess the impact of an MI training program on 222 corrections employees who were mandated to attend five-days of training. Fair to excellent inter-rater reliability estimates were reported across both VASE-R and WRQ scores, and acceptable internal consistency was found for the WRQ. In another study (Doran et al., 2013), the WRQ was used to assess the effectiveness of an MI training program on 1,552 correctional staff. Consistent with earlier findings, the WRQ showed fair to excellent levels of inter-rater reliability and significant MI skills gains were documented following three days of MI training. Finally, in a demonstration project examining the effectiveness of an MI training curriculum, 23 officers from a large probation department completed both the ORQ and were coded on the MITI (Walters et al., 2010). Scores on the ORQ and MITI were highly correlated, and officers showed patterns of improvements on both tools following MI training.

Although generally less labor-intensive than observation tools, practitioner completed tools also require expert coding of written material. In the studies reviewed, little information was provided regarding the training procedures of coders/raters, as well as the time needed to score a practitioner’s written responses. Thus, time requirements for implementing these tools in criminal justice settings remains unclear.

### Other assessment strategies

Several other assessments strategies are worth noting. Designed as a measure of MI *knowledge* rather than skill, the Motivational Interviewing Knowledge and Attitudes Test (MIKAT; Leffingwell, 2006) is a 29-item tool used to assess the educational impact of training. About half of the items are presented in a true/false format, while the remaining items list interaction styles (e.g., express empathy, give direct advice) and respondents are asked to check the items that are consistent with the MI approach. Higher scores reflect greater MI knowledge. In studies with criminal justice practitioners, the MIKAT has demonstrated good levels of internal consistency, as well as sensitivity to the impacts of training (Hohman et al., 2009; Doran et al., 2013).

Originally developed by Madson et al. (2009), the Client Evaluation of Motivational Interviewing (CEMI) is a *client self-report* tool designed to assess clients’ perceptions of MI use by practitioners (i.e., from the client’s perspective). A probation version was validated by Armstrong et al. (2016) with a sample of 485 probationers in an office where all officers received MI training. The CEMI-Probation version takes about 15 min to complete, and 15 items are measured on a 4-point Likert-type scale with responses ranging from 1 (never) to 4 (a great deal) reflecting both technical and relational aspects of MI. Factor analytic support was found for both the relational and technical subscales, as well as acceptable-to-very good levels of internal consistency. The CEMI-Probation version has not been compared with more established practitioner observation tools and the relationship with client recidivism has not been explored.

Modeled after the readiness ruler used by Heather et al. (2008), the Quick Readiness Measure (QRM) consists of a one or two items asking practitioners to self-rate their level of *motivation to utilize MI* and/or *perceived effectiveness of MI* with their cases. Items are rated on a scale from 1 (not at all) to 10 (extremely). Mixed findings with criminal justice practitioners have been found on the QRM in terms of sensitivity to pre-post training effects (Hohman et al., 2009; Doran et al., 2013).^2^

Finally, technology has recently been utilized to make coding MI sessions more efficient. Typically, conversations are converted into transcripts which are then analyzed to count specific responses according to algorithmic decision rules (Klonek et al., 2015; Tanana et al., 2016; Imel et al., 2019). *Technology assisted coding* shows great promise for increasing consistency and reducing time, however, such models have not yet been adapted to criminal justice environments.

## Present research

In considering the MI skills assessment literature in general, *practitioner rating tools* emerge with the most empirical evidence. However, the selection of a tool depends on the goals of assessment (e.g., to evaluate the impact of training initiatives, identify practitioners who need additional support, etc.) and several practical issues (e.g., number of practitioners being assessed, resources to train assessors and code work samples). In fast-paced, criminal justice environments, assessment strategies that are time-efficient and inexpensive are usually preferred. The present article describes the development and initial validation of the Response Style Screening Questionnaire (RSSQ), a new practitioner completed tool to assess an *MI-consistent practice orientation*. The rationale for developing a new tool was to create an assessment that does not require coding of either recorded work samples or written practitioner responses.

This article presents data from three studies that examined the factor structure and correlates of the RSSQ. *Study 1* describes item and subscale scale development and an exploratory factor analysis. In *Study 2*, we conducted a confirmatory factor analysis and a preliminary analysis of construct validity by examining the RSSQ’s relationship with self-reported measures of probation officer (PO) attitudes and behaviors. In *Study 3*, our analysis of the validity of the RSSQ was extended to observable PO behavior by examining the correlates of the screening tool with MI and core correctional practices (CCPs) in a sample of audio recorded PO-client office visits, as well using the RSSQ to assess the impact of training.

Several hypotheses guided this initial validation phase. First it was hypothesized that, similar to other instruments, the RSSQ would have a multidimensional factor structure revealing both MI-consistent and MI-inconsistent practitioner response styles. Second, it was hypothesized that MI-consistent response styles would be more strongly associated with a “change-agent” philosophy among probation officers and intrinsic work motivation to help probationers succeed, while MI-inconsistent response styles would be linked to an enforcement/compliance mindset and extrinsic work motivation. Third, it was predicted that practitioners with MI-consistent styles of responding would show greater utilization of agency resources designed to help probationers, whereas those with MI-inconsistent styles would report lower levels of utilization. Fourth, MI-consistent response styles would be correlated with greater skillfulness displayed during actual office visits. Alternatively, MI-inconsistent response styles would be inversely related to observed skills. Fifth, based on prior studies examining the effects of training, it was expected that practitioners’ RSSQ scores would show a pattern of improvement following training experiences.

## Study 1: scale development and exploratory factor analysis

### Development of the RSSQ

The RSSQ is organized around five brief case scenarios and hypothetical client statements that might be made to a forensic practitioner (see Table 2). While similar to the approach described for other practitioner completed tools (e.g., HRQ, ORQ, and WRQ), RSSQ case scenarios are novel except for the first one which was adapted from the ORQ. For each scenario, respondents are asked to imagine they are in the beginning phase of working with the client. Unlike the open-ended format of other practitioner completed tools, RSSQ case stimuli are followed by a series of potential practitioner responses. Respondents are then asked to rate on a 4-point Likert-type scale the degree to which each of the potential responses is consistent with their style of reacting to a client in that situation (1 = never, not at all consistent with my natural style; 2 = rarely, not typical of my style; 3 = sometimes, somewhat consistent with my style, could see myself saying something like this with certain people; 4 = frequently, very close to my natural style). If respondents have not encountered a situation similar to a case scenario, they are asked to imagine how they would naturally respond.

In the initial piloted version of the RSSQ, each case stimulus was followed by five standard potential practitioner responses (items) designed to be indicative of a different style of interaction ranging from MI-inconsistent to MI-consistent. Process research has supported the hypothesis that practitioner MI-inconsistent skills are linked with greater client sustain talk, and more sustain talk is associated with worse outcomes; whereas MI-consistent skills are associated with a higher proportion of client change talk, and more change talk is associated with better outcomes (Magill et al., 2018). Thus, items were conceptualized around the types of verbalizations that a response would likely to produce from the client (change talk, sustain talk/discord). Below is a description of the five response styles and sample items related to one of the case scenarios:

1. *Confrontational* style responses are likely to produce discord, increase client resistance, and have the potential to be harmful (e.g., *“First of all, watch your tone and do not swear in my office. You’re not talking to your girlfriend now; you are talking to me.”*).
2. *Sustain Talk* style responses are likely to elicit sustain talk and may inadvertently reinforce helplessness or justifications for not changing (e.g., “*Sounds like she is to blame for most of what has happened.*”).
3. *Neutral* style responses are likely to result in limited client verbalizations and are a mix of closed questions and advice statements (e.g., “*However all this came about, you need to make the best of it now. Just do what the judge required, and you can put all this behind you.”*).
4. *Eliciting* style responses are likely to increase client verbalizations and elicit new information (e.g., “*What kinds of things do you argue about*?*”*).
5. *Change Talk* style responses focus on the parts of client statements most related to change and are likely to evoke change talk and reinforce motivation for change (e.g., “*Sounds like you have some real concerns over how all this arguing is affecting your kids.”*).

The original conceptualization of the RSSQ was designed to yield scores on 5 subscales (one for each response style). The stimuli and responses were developed by a clinical psychologist and a retired probation officer who both have extensive experience training criminal justice practitioners, are members of the MI Network of Trainers (MINT), and published articles and book chapters on the topic of MI in forensic practice. See Tafrate et al. (2023) in the Supplementary material for the final RSSQ items, instructions, and benchmarks for interpretation.^3^

### Participants and procedure

Eight hundred and twenty-five criminal justice practitioners (e.g., case managers, correctional counselors, POs) served as the sample for the exploratory factor analysis (EFA) of the RSSQ. Participants were attending MI training workshops as part of their ongoing professional development. Overall, 20.4% (*n* = 168) had no prior training in MI, while the majority reported at least some prior exposure to the MI model. Practitioners completed the RSSQ along with demographic information at the beginning of their workshop. Table 1 provides demographic data on participants in all three studies. All three studies were approved by the Institutional Review Board of the authors’ university.

**Table 1:** Demographic characteristics of participants.

Variable	Study 1 *N* = 825	Study 2 *N* = 350	Study 3 *N* = 33
Age			
21–30 years	52% (*n* = 429)	15.1% (*n* = 53)	12.1% (*n* = 4)
31–40 years	21.7% (*n* = 179)	54% (*n* = 189)	42.4% (*n* = 14)
> 40 years	23.9% (*n* = 197)	29.1% (*n* = 102)	42.4% (*n* = 14)
Missing	2.4% (*n* = 20)	1.7% (*n* = 6)	3% (*n* = 1)
Gender			
Female	64.5 (*n* = 532)	52.6% (*n* = 184)	60.6% (*n* = 20)
Male	34.9 (*n* = 288)	45.1% (*n* = 158)	39.4% (*n* = 13)
Missing	0.6% (*n* = 5)	2.3% (*n* = 8)	0
Ethnicity			
White	48.7% (*n* = 402)	58% (*n* = 203)	63.6% (*n* = 21)
Black	23.0% (*n* = 190)	18.9% (*n* = 66)	12.1% (*n* = 4)
Latino/a	20.2% (*n* = 167)	13.7% (*n* = 48)	12.1% (*n* = 4)
Asian American	1.0% (*n* = 8)	0.6% (*n* = 2)	0
Other	5.1% (*n* = 42)	4.3% (*n* = 15)	6.1% (*n* = 2)
Missing	1.9% (*n* = 16)	4.6% (*n* = 16)	6.1% (*n* = 2)

### Results

The EFA of the RSSQ was examined with the SPSS principal axis factoring option with a varimax rotation. EFA was chosen over principal components analysis as we had *a priori* expectations about how the variables would be related (i.e., specific response styles) (Costello and Osborne, 2005). Parallel analysis was used to determine the number of factors to be retained as the procedure has been found to be less likely than standard methods to result in the retention of spurious factors (Thompson, 2004). In parallel analysis, eigenvalues derived from the factor analysis are compared with eigenvalues derived from a randomized sorting of the data. Only those factors whose factor analytic derived eigenvalues are greater than those derived from randomized sorting are retained (O’Connor, 2000). Items were retained on a factor if they loaded >0.40, did not cross-load greater than .32 on a second factor, and cross loadings on other factors were at least .20 lower than on the primary factor (Tabachnick and Fidell, 2001).

**Figure 1:**
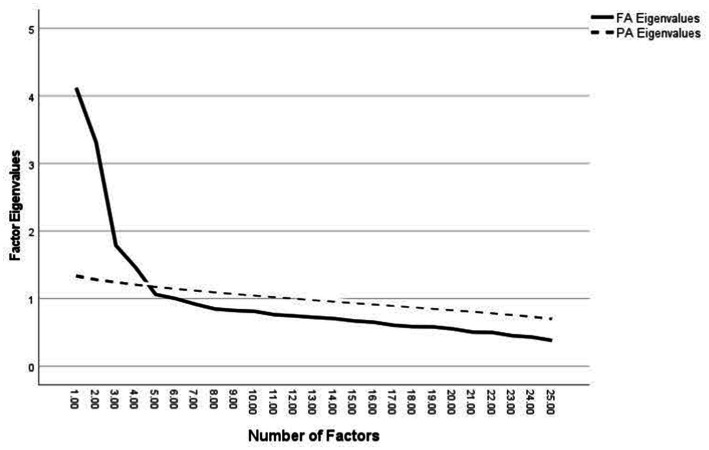
Exploratory factor analysis scree plot. FA, factor analysis eigenvalues; PA, parallel analysis eigenvalues.

A Kaiser-Meyer-Olkin value of 0.83, and a significant Bartlett’s test of sphericity (*p* < 0.0001) indicated the dataset was appropriate for factor analysis. The EFA with parallel analysis yielded four factors whose eigenvalues exceeded their randomly ordered counterparts (see Figure 1). Factor 1 was consistent with the Confrontational style and contained 6 items (five of which had been originally designated for that subscale and one item that had previously been assigned to the neutral subscale). Factor 2 was consistent with the Sustain Talk style as it contained 4 items, all of which had been conceptually designated for that subscale. Factor 3 was consistent with the Eliciting style and contained 4 items (three of which had been originally designated for that subscale and one that had previously been created for the neutral subscale). Factor 4 was consistent with the Change Talk style as it contained 4 items, all of which had been conceptually designated for that subscale. Table 2 contains a summary of the factor loadings from the EFA. The superiority of the resulting 18 item 4-factor model over the originally planned 25 item 5-factor model was subsequently tested in Study 2 with confirmatory factor analyses. Initial validity indicators of the RSSQ were also examined in Study 2.

**Table 2:** Summary of items and exploratory factor analysis item loadings.

Client Examples/Statements	Confrontation	Sustain talk	Eliciting	Change talk
*Scenario 1*: A 22-year-old man tells you: *“I want to stay clean and sober, but I cannot afford to get my own place yet; so, I have to live with my brother who drinks all the time.”*				
1a. “The bottom line is if you start using again, you are going to get violated. I do not know how you are going to handle this, but you need to remember that your brother is not going to be doing your time for you.”	**0.62**	0.08	0.01	−0.07
1b. “Part of you really wants to stay clean, but the situation with your brother makes that difficult.”	−0.06	0.02	−0.01	**0.42**
1c. “It sounds like if you start using again, it will definitely be because of your brother’s negative influence.”	0.04	**0.41**	0.09	−0.01
*Scenario 2*: A 39-year-old man tells you: *“I’m not a criminal, I’m an addict. You do not understand what it is like. It is not like I can just stop. I know you just want to keep taking urines on me so I go to jail. You need to help me.*”				
2a. “It seems like you are really looking for some help at this point.”	−0.13	−0.04	0.12	**0.56**
2b. “Sounds like you are an addict, and it would be almost impossible for you to change.”	0.13	**0.46**	−0.05	−0.05
2c. “When you choose to break the law there are consequences. Drug use is a choice. If you end up in jail it will not be because of me. It will be because you made the wrong choices.”	**0.63**	0.04	0.01	−0.10
*Scenario 3*: A 44-year-old man tells you: *“You’re damn right I’m angry. The system is unfair to men. She should be here and not me. She starts all the arguing and does not know when to stop. The sad thing is that my kids are affected by all this fighting and drama.”*				
3a. “What kind of things do you argue about?”	0.16	0.12	**0.65**	0.01
3b. “Sounds like you have some real concerns over how all this arguing is affecting your kids.”	−0.07	−0.06	0.20	**0.63**
3c. “Sounds like she is to blame for most of what has happened.”	0.15	**0.66**	0.11	0.05
3d. “However all this came about, you need to make the best of it now. Just do what the judge required, and you can put all this behind you.”	**0.62**	0.04	0.08	−0.16
3e. “First of all, watch your tone and do not swear in my office. You’re not talking to your girlfriend now; you are talking to me.”	**0.49**	0.11	−0.03	−0.03
*Scenario 4*: A 24-year-old woman tells you: *“I know I sometimes make poor decisions. I’ve had a lot of things happen to me; there’s things that bother me. I get depressed. I get emotional just talking about it. But I do not think I’m crazy. I’m not sure about counseling. I do not see how talking about my problems will help anything. It will probably make me feel worse.”*				
4a. “There’s no way of knowing how you’ll feel until you have tried it. In any event, counseling is required as part of your probation. You will not feel very good if you end up back in court on a violation.”	**0.67**	0.03	0.04	−0.02
4b. “It seems like you would not be able to handle going to counseling.”	0.09	**0.56**	0.06	0.08
4c. “Tell me more about your history of depression.”	−0.06	0.04	**0.55**	0.27
*Scenario 5*: A 52-year-old man tells you: *“I do not really think I should be here. I’m not an abuser. It was just an argument that got out of hand.”*				
5a. “Tell me more about what led up to the argument.”	−0.07	0.01	**0.67**	0.18
5b. “I’ll bet if we took a poll of everyone else sitting in the waiting room, none of them think they should be here either. This program is not voluntary. It’s a court order.”	**0.59**	0.21	−0.04	0.02
5c. “How long have you been in this relationship?”	0.10	0.11	**0.67**	0.08
5d. “You sound concerned and embarrassed that this argument got so out of hand.”	−0.01	0.09	0.15	**0.42**

Items in bold loaded on the factor indicated at the top of the column.

## Study 2: confirmatory factor analysis and initial construct validation

Study 2 consisted of a statewide survey of adult POs focused on their perceptions regarding aspects of their role and work. Survey responses were used to conduct a confirmatory factor analysis that tested the 4-factor RSSQ model against the original 5-factor RSSQ model.

### Participants and procedure

The surveys were administered in every adult probation office across the state during weekly staff meetings. A total of 350 completed surveys were collected, representing an 80% response rate. In addition to the demographic information provided in Table 1, 25% of POs had more than 10 years on the job, 27% had 7 to 10 years, 34% had 4 to 6 years, and 13% had less than four years.

### Materials

In addition to the RSSQ, the survey included several scales related to POs’ supervision orientation, work-related motivations, and appraisals of the frequency of use and usefulness of various supervision resources provided by their agency.

#### Probation officer supervision orientation

Probation officers were asked to identify how they approach their jobs using an adapted version of Shearer’s (1991) Probation Strategies Questionnaire (PSQ). This instrument provided POs with 24 statements regarding their roles and supervision strategies and asked them to rate how well each described their approach using a 6-point Likert scale. The items measured POs role identification based on three predominant probation strategies: law enforcement or compliance-based (POs view their role as monitoring probationers’ compliance with the rules of the probation sentence), resource broker or referral-based (POs primarily refer probationers to treatment and/or employment programs), and casework or counseling-based (POs view themselves as change agents whose role is to help their probationers address personal problems). The internal and external reliability and validity of the PSQ has been supported in previous studies (Sluder et al., 1991; Shearer, 2002). In the current sample, internal consistency was in the acceptable range for the casework/counseling (*α* = 0.78) and broker/referral (*α* = 0.74) subscales, and in the questionable range for enforcement/compliance (*α* = 0.66).

#### Intrinsic and extrinsic work motivation

Employees’ motivation to perform their work-related duties is believed to center on their intrinsic and extrinsic reasons (Van de Broech et al., 2021). Intrinsic motivation consists of performing an activity out of inherent interest or pleasure, whereas extrinsic motivation is based on the idea that people are motivated by a separable outcome such as external rewards and avoiding punishments (Ryan and Deci, 2017).

We measured work motivation using Tremblay et al.’s (2009) Extrinsic and Intrinsic Motivation Scale. This instrument is comprised of 18 statements where respondents are asked how much each statement corresponds to why they perform their work-related responsibilities. The responses are measured on a 7-point Likert scale ranging from “Does not correspond at all” to “Corresponds exactly.” These 18 items are divided into six 3-item subscales that represent different aspects of intrinsic and extrinsic motivation. These are: *intrinsic motivation* (doing an activity for its own sake because a person finds it interesting and satisfying), *integrated regulation* (valuing an activity to where it regularly enhances a person’s self-worth), *identified regulation* (doing an activity because one finds value or meaning in it), *introjected regulation* (regulating a behavior based on how much it positively or negatively effects self-worth), *external regulation* (doing an activity to receive an external reward), and *amotivation* (a lack of motivation or desire to act passively). In addition to these six subscales, the 18 items are used to create an overall *Self-Determination Index,* which represents how much employees approach their jobs in a way that they find inherently interesting and satisfying. Previous studies in criminal justice populations have shown this measure to have good internal reliability and construct validity (Breaugh et al., 2017). The internal consistencies of the subscales in the current sample were mostly in the acceptable range (*α* = 0.72 to .80), except for identified regulation (*α* =0.46) and introjected regulation (*α* =0.67).

#### Frequency of use and perceptions of usefulness of probation supervision resources

POs across the state have access to several types of probation resources to help them better supervise their clients. We created this component of the survey to explore how often POs report using these resources and their perceptions of their usefulness. The *frequency of use* scale ranged from 1 (never use) to 4 (often use) while the *perception of usefulness* items ranged from 1 (not useful) to 4 (very useful). The list of probation resources included motivational interviewing, the Level of Service Inventory-Revised (a risk/needs assessment), the Adult Substance Use Survey-Revised (a client self-report of substance use), Probationer Risk Assessments Report (a summary of clients risk/needs assessment histories), Case Plan Report (shows case plan and client progress), and the What I Want to Work on Questionnaire (a questionnaire where probation clients can identify personal issues they want to address during their supervision).

### Results

#### Confirmatory factor analyses

CFAs were conducted with EQS for Windows 6.4. The 4-factor model was tested against the original 5-factor model for comparison purposes. We used the Maximum Likelihood with Robust Methods due to the ordinal nature of the items (Li, 2015). We evaluated model fit with the Comparative Fit Index (CFI), Standardized Root Mean Residual (SRMR), and Root Mean Square Error of Approximation (RMSEA). As a guideline, models producing CFI values greater than .90, SRMR values below 0.08, and RMSEA values below .06 are considered a good fit (Hu and Bentler, 1999).

As summarized in Table 3, the CFI for the four-factor model was 0.91, the SRMR was 0.06, and the RMSEA was .05, indicating an adequate fit. On all three indicators, the four-factor model provided a better fit than the five-factor model. The improvement in fit yielded by the four-factor model over the five-factor model was evaluated by subtracting the chi-square value for the four-factor model from the chi-square value for the five-factor model, subtracting the degrees of freedom for the four-factor model from the degrees of freedom for the five-factor model, and evaluating the resulting Δχ^2^ as an ordinary chi-square (Thompson, 2004). This procedure indicated that the four-factor model provided a significant improvement in fit over the five-factor model, Δχ^2^ = 304.93, Δ*df* = 136, *p* < 0.001.

**Table 3:** Summary of confirmatory factor analyses.

Model	χ^2^	*df*	CFI	SRMR	RMSEA	RMSEA 90% CI
Four-factor model	234.60	129	0.91	0.06	0.05	[0.04, 0.06]
Five-factor model	539.53	265	0.83	0.09	0.06	[0.05, 0.06]
Second-order model	174.99	107	0.96	0.04	0.04	[0.32, 0.06]

CFI, Comparative Fit Index; CI, confidence interval; SRMR, Standardized Root Mean Residual; RMSEA, Root Mean Square Error of Approximation.

To further examine the structure of the instrument with regard to overarching MI inconsistent-consistent styles, we conducted a second-order confirmatory factor analysis with the Confrontational and Sustain Talk styles hypothesized on one factor and the Eliciting and Change Talk styles on another factor. As summarized in Table 3, this model fit the data well and suggests that the RSSQ corresponds with the conceptualization of practitioner skills as falling broadly into those that are MI-inconsistent versus MI-consistent (Magill et al., 2018).

#### RSSQ descriptive statistics

Descriptive statistics for the RSSQ, including intercorrelations, are presented in Table 4. Subscale items were averaged to produce subscales scores. As expected, scores on MI-inconsistent response styles likely to produce discord or reinforce reasons for not changing were significantly correlated with each other (i.e., Confrontational and Sustain Talk styles), whereas scores on MI-consistent response styles likely to encourage client verbalizations and reinforce motivation for change were significantly correlated (i.e., Eliciting and Change Talk styles). The Confrontational style was also negatively correlated with the Change Talk style. Cronbach’s alphas and McDonald’s omegas for three of the subscales were above 0.70, while the Sustain Talk style coefficients approached 0.70.

**Table 4:** RSSQ descriptive statistics.

Subscale	Cronbach’s α	McDonald’s omega	*M*	*SD*	1	2	3	4
1. Confrontational style	0.75	0.75	1.92	0.59	–			
2. Sustain talk style	0.68	0.68	1.60	0.56	0.25***	–		
3. Eliciting style	0.78	0.78	3.23	0.58	0.02	0.08	–	
4. Change talk style	0.74	0.74	3.35	0.59	−0.12*	0.06	0.53***	–

^*^p < 0.05. ^***^p < 0.001.

#### Correlates of the RSSQ: patterns of convergence and divergence with an MI orientation

The correlations between the work perception measures and the RSSQ constructs are presented in Table 5. As predicted, endorsement of an enforcement/compliance-based probation strategy was associated with a Confrontational style, and negatively correlated with a Change Talk style. The broker/referral agent and case work/counselor role perceptions were associated with both the Eliciting and Change Talk styles. The case work/counselor orientation was also negatively correlated with the Confrontational style. The Sustain Talk style was unrelated to POs’ reported supervision strategy.

**Table 5:** RSSQ correlations with probation officer orientation and work motivation.

	Confrontational style	Sustain talk style	Eliciting style	Change talk style
Probation supervision orientation				
Enforcement/Compliance	0.27^**^	0.01	−0.06	−0.16^**^
Broker/Referral	−0.04	−0.07	0.28^**^	0.31^**^
Case work/Counseling	−0.12^*^	0.07	0.38^**^	0.39^**^
Work motivation				
Intrinsic motivation	−0.11^*^	0.03	0.24^**^	0.25^**^
Integrated regulation	0.07	−0.01	0.18^**^	0.17^**^
Identified regulation	0.10	0.05	0.21^**^	0.22^**^
Introjected regulation	−0.01	−0.001	0.15^**^	0.11^*^
External regulation	0.15^**^	−0.02	0.09	0.09
Amotivation	0.29^**^	0.13^*^	0.04	−0.06
Self-determination index	−0.23^**^	−0.04	0.11^*^	0.19^**^

*p < 0.05. **p < 0.01. ***p < 0.001.

Similar patterns emerged for the work motivation subscales and the RSSQ (Table 5). POs with a Confrontational style scored higher on External Motivation and Amotivation, while scoring lower on Intrinsic Motivation and the overall Self-Determination Index. Alternatively, POs scoring higher for Eliciting and Change Talk styles had significant positive correlations with all four of the intrinsic motivation subscales, as well as the Self-Determination Index. The Sustain Talk style was generally unrelated to job motivation, except for a significant correlation with Amotivation.

Correlations between the RSSQ and the frequency of use and perceptions of usefulness of various supervision resources are shown in Table 6. For *frequency of use*, the Confrontational style was correlated with the utilization of risk/needs assessment and none of the other resources, while Sustain Talk style was not correlated with the use of any resources. As expected, POs scoring high on Elicit and Change Talk styles reported more frequent use of motivational interviewing skills, agency reports of clients’ risk assessment scores and case plans, and the What I What to Work on Questionnaire. POs who scored higher on the Eliciting style also reported more use of the Adult Substance Use Survey.

**Table 6:** RSSQ correlations with probation officer frequency of use and perceptions of usefulness of probation supervision resources.

	Confrontational style	Sustain talk style	Eliciting style	Change talk style
Frequency of use				
Motivational interviewing	−0.05	07	0.29^**^	0.40^**^
Level of Service Inventory-Revised	0.12^*^	0.05	0.10	0.08
Adult Substance Use Survey-Revised	0.03	0.07	0.15^**^	0.07
Probationer Risk Assessments Report	0.07	0.11	0.12^*^	0.15^**^
Case Plan Report	0.08	0.07	0.12^*^	0.13^*^
What I Want to Work on Questionnaire	0.04	0.10	0.18^**^	0.18^**^
Perception of usefulness				
Motivational interviewing	−0.19^**^	0.02	0.23^**^	0.41^**^
Level of Service Inventory-Revised	−0.13^*^	0.10	0.24^**^	0.32^**^
Adult Substance Use Survey-Revised	−0.14^**^	0.02	0.19^**^	0.28^**^
Probationer Risk Assessments Report	−0.03	0.09	0.21^**^	0.29^**^
Case Plan Report	−0.10	0.09	0.15^**^	0.26^**^
What I Want to Work on Questionnaire	−0.12^*^	0.14^*^	0.16^**^	0.23^**^

^*^p < 0.05. **p < 0.01. ***p < 0.001.

The patterns of correlations were stronger for *perceptions of usefulness*. A Confrontational style was negatively correlated with POs’ ratings of usefulness for motivational interviewing, Level of Service Inventory, the Adult Substance Use Survey, and the What I want to Work on Questionnaire. In contrast, POs scoring higher on Eliciting and Change talk response styles were consistently more likely to see value in all the agency resources. The Sustain Talk style was generally unrelated to perceptions of usefulness, except for a significant positive correlation with the What I Want to Work on Questionnaire.

## Study 3: preliminary criterion-related validity

In Study 3, the relationship between the RSSQ and practitioner behavior with clients was examined. Using audio recorded work samples of PO-client office visits, the quality of MI skills and CCPs were coded and correlated with the RSSQ. CCPs are derived from a body of empirical work identifying effective PO intervention practices and include relational skills, modeling, reinforcement, guiding sessions in a productive manner, and cognitive restructuring (Andrews and Kiessling, 1980; Dowden and Andrews, 2004; Taxman, 2008; Trotter, 2013).

Since many of the previously reviewed tools have shown sensitivity to the effects training, pre-to-post training changes on the RSSQ were also examined after completion of a cognitive-behavioral therapy (CBT) program that incorporated CCPs and elements of MI. Due to the small sample size, effect sizes rather than *p* values were used to guide the interpretation of these findings. Similarly, we reexamined the sample in Study 1 to see if there were RSSQ differences in participants who had prior exposure to MI and those whose attendance at training marked their first exposure to the MI model.

### Participants

Participants were thirty-three POs attending a training program to use CBT techniques in office visits with their clients (see Table 1 for demographic information). All officers were supervising medium to high-risk cases. In terms of experience as a PO, 81.8% (*n* = 27) had served over 10 years, while 12.1% (*n* = 4) had served between 1 and 9 years, and 6.1% (*n* = 2) had served less than a year in the PO role. All officers had undergone MI training when first hired.

### Materials and procedure

POs completed the RSSQ prior to starting the CBT training program and again upon completion. Also prior to starting the program, POs were asked to submit work samples consisting of three audio recordings of office visits with medium or high-risk clients. A sample of 113 codable recordings out of an expected 117 were obtained (one officer did not submit any recordings and another submitted two rather than three). The average length of the recordings was 13.93 min (*SD* = 9.70). Audio recordings were coded for specific MI and CCPs skills. Coding was conducted by a team of trained coders blind to the identity of the POs and clients. After completion of coding training, intraclass correlation coefficients for individual variables ranged from 0.73 to 0.99. Each recording was analyzed by two coders, who worked independently, and then compared scores afterward to produce a consensus coding. Remaining scoring disagreements were resolved through discussion with a senior coder. Twenty-five randomly selected recordings were double coded by an additional pair of coders for reliability analyses, which yielded an average intraclass correlation coefficient of 0.80.

#### MI skills

For each recording, coders rated the proficiency of the PO on four MI skills: (1) *Reflections*, (2) *Open-ended questions*, (3) *Affirmations*, and (4) *Evoking change talk*. Each skill was rated on a 3-point scale ranging from −1 to +1. Coders were provided with behavioral descriptions of each anchor point.

#### CCPs skills

For each recording, coders rated the proficiency of the PO on three CCPs skills (1) *Empathy*, (i.e., the extent to which the officer made efforts to understand the client’s perspective), (2) *Collaborative spirit* (i.e., the extent to which the PO approached the client in a collegial versus adversarial manner, and worked toward mutually agreed upon goals), and (3) *Constructive use of authority* (i.e., the extent to which the officer appeared to be guiding session in a purposeful and productive manner). As with MI skills, CCPs were rated on 3-point scale ranging from −1 to +1, with behavioral descriptors provided for each anchor point.

### Results

#### Relationship between RSSQ, MI, and CCPs

As summarized in Table 7, scores on the RSSQ were largely correlated in expected directions with coded work samples of MI Skills and CCPs. Regarding *MI skills*, the Confrontational style was negatively correlated with all 4 MI skills (with small to moderate effect sizes), while Sustain Talk was also negatively associated with MI skills (with small effect sizes). Surprisingly, Eliciting was unrelated to MI skills. The Change Talk style was positively correlated with all 4 MI skills with effect sizes in the moderate range.

**Table 7:** RSSQ correlations with coded motivational interviewing skills and core correctional practices.

Subscale	Reflect	Open	Affirm	Evoke CT	Empathy	Collab spirit	Construct AU
Confrontational style	−0.25	−0.24	−0.45*	−0.32^†^	−0.22	−0.34^†^	−0.22
Sustain talk style	−0.17	−0.23	−0.27	−0.22	−0.23	−0.04	0.04
Eliciting style	−0.17	−0.16	0.05	−0.17	−0.04	0.06	0.22
Change talk style	0.33^†^	0.32^†^	0.33^†^	0.30^†^	0.37*	0.38*	0.30^†^

Reflect, Reflections; Open, Open ended questions; Affirm, Affirmations; Evoke CT, Evoking change talk; Empathy, Empathy; Collab Spirit, Collaborative spirit; Construct AU, Constructive use of authority. ^†^*p* < 0.10. ^*^p < 0.05.

With respect to *CCPs*, the Confrontational style had small to moderate negative correlations with all 3 CCPs, while Sustain Talk style had a small to moderate negative correlation with Empathy. Eliciting had a small to moderate positive correlation with Constructive Use of Authority, and the Change Talk style had positive correlations with all 3 CCPs in the moderate range.

#### The relationship between training and the RSSQ

Changes in RSSQ scores from a pre-to-post CBT training were analyzed with t-tests, controlling for alpha with a Bonferroni correction procedure (0.05/4 = 0.0125), while also examining effect sizes (Cohen’s *d*), given the small sample size. As summarized in Table 8, scores on the Confrontational style subscale moved in the direction of improvement, significantly decreasing with a moderate effect size. Scores on the Eliciting and the Change Talk scales also moved in the direction of improvement (with small effect size increases), with significant improvement noted for Eliciting. The Sustain Talk style did not show any training related changes.

**Table 8:** Changes in RSSQ scores from pretraining to posttraining.

Subscale	Pretraining		Posttraining					
	*M*	*SD*	*M*	*SD*	*t*	*p*	Cohen’s *d*	95% CI
Confrontational style	2.02	0.50	1.74	0.48	−3.03	0.005	−0.54	[−0.92, −0.16]
Sustain talk style	1.51	0.46	1.52	0.44	0.19	0.851	0.03	[−0.32, 0.37]
Eliciting style	3.40	0.57	3.56	0.47	2.08	0.046	0.37	[0.01, 0.74]
Change talk style	3.28	0.68	3.42	0.49	1.54	0.133	0.28	[−0.08, 0.64]

*N* = 31. df = 30. CI, confidence interval.

Given the changes in the RSSQ associated with the CBT training program, the sample in Study 1 was reexamined for RSSQ differences between participants who reported no prior MI training and those who indicated previous MI training (see Table 9). Similar to the patterns observed in the sample from Study 3, participants with prior MI training scored significantly lower on the Confrontational style and significantly higher on the Change Talk style, with differences consistent with small to moderate effect sizes. The Eliciting style was also in the right direction with a significant improvement (but with a small effect size), while the Sustain Talk scale once again revealed almost no training effect.

**Table 9:** Differences in RSSQ scores between practitioners with and without prior MI training.

Subscale	No Prior MI (*n* = 168)		Prior MI (*n* = 657)					
	*M*	*SD*	*M*	*SD*	*t*	*p*	Cohen’s *d*	95% CI
Confrontational style	1.91	0.62	1.66	0.58	4.87	<0.001	−0.42	[−0.59, −0.25]
Sustain talk style	1.39	0.40	1.49	0.51	−2.24	0.025	0.19	[0.02, 0.36]
Eliciting style	3.29	0.58	3.39	0.57	−2.05	0.041	0.18	[0.01, 0.35]
Change talk style	3.34	0.49	3.50	0.45	−4.18	<0.001	0.36	[0.19, 0.53]

*N* = 825. df = 823. CI, confidence interval.

## Discussion

The purpose of this project was to validate a new practitioner completed tool to assess an MI practice orientation. The RSSQ differs from other common assessments of MI competence in that it does not require coding of recordings or ratings of written practitioners’ responses. Our intention was to create a screening tool that could be practically implemented in criminal justice environments and that would not require confidential work samples, labor-intensive training of evaluators, and complicated scoring. The RSSQ takes approximately five minutes to administer and five minutes to score. Data from the three studies provide initial support for the factorial, construct, and criterion validity of the RSSQ. Results are discussed in terms of the specific hypotheses tested, implications for criminal justice agencies and programs, as well as limitations and future directions.

### Hypotheses

As predicted in the first hypothesis, the findings reveal a multidimensional model of MI practice orientation. Studies 1 and 2 resulted in an 18-item, four factor tool with acceptable internal consistency across the four scales (i.e., response styles). Two of the RSSQ styles correspond with MI-consistent practitioner behaviors (Change Talk and Eliciting), while two styles correspond with MI-inconsistent practices (Sustain Talk and Confrontational). The pattern of the scales’ intercorrelations and the second-order factor structure provide additional evidence for the MI-consistent versus inconsistent conceptualization, similar to several assessment tools reviewed earlier (e.g., MISC, MITI, VASE-R, MIKAT).

With respect to the second hypothesis, correlations between the RSSQ constructs and work perception measures revealed that POs with MI-consistent response styles viewed their work differently than those with MI-inconsistent styles. Both MI-consistent response styles were significantly correlated with a “change agent” view of the PO role—directly fostering client behavior change and making referrals to interventions. Not surprisingly, the MI-inconsistent Confrontational style was aligned with a probation approach centered around enforcement, surveillance, and compliance with court mandated requirements. The Sustain Talk style was not significantly linked to any specific probation philosophy.

Similarly, as hypothesized, POs who endorsed both MI-consistent response styles reported higher levels of intrinsic motivation (i.e., wanting to do a good job for the inherent satisfaction they would feel). Alternatively, officers possessing the more extreme Confrontational style tended to be motivated by external rewards (e.g., paycheck, avoiding criticism) or reported being minimally engaged in the job. The overall pattern of correlations between RSSQ and the work perception and attitude measures provide support for the convergent and divergent validity of the RSSQ and adds to the small body of literature on PO job-related views and potential client outcomes (Skeem et al., 2007; Bourgon, 2013; Viglione et al., 2017; Lovins et al., 2023).

As predicted in the third hypothesis, and further supporting the validity of the RSSQ, POs endorsing both MI-consistent response styles were more likely to embrace and utilize agency resources when working with their clients. In contrast, almost no relationship was found between MI-inconsistent styles and reported resource utilization. Moreover, in considering reported usefulness of available resources, officers exhibiting the more extreme Confrontational style seemed to perceive little value in using them. Once again, POs with both MI-consistent response styles viewed their job activities differently than their counterparts who were less MI-adherent.

Given the importance of actual PO behavior as a validation criterion, the fourth hypothesis predicted a specific pattern of correlations between the response styles measured on the RSSQ and recordings of PO-client interactions coded for MI skills and CCPs. As expected, the Change Talk style was moderately correlated with all four MI skills (reflecting, open questions, affirming, and evoking change talk). Likewise, a uniform pattern of small to moderate negative correlations was found between the Confrontational and Sustain Talk styles and all four MI skills. The notable exception to our predicted pattern of correlations was the negligible relationship between the Eliciting style and MI skills. POs in Study 3 tended to score high on Eliciting (averaging over a 3 out of 4 on the scale) even before exposure to training. Thus, we speculate a ceiling effect may have contributed to this scale not performing in a way that was consistent with the original hypothesis.

Regarding the CCPs skills of empathy, collaboration, and constructive use of authority, the strongest patterns of correlations emerged for RSSQ scales representing the more extreme styles. For example, the Change Talk style uniformly produced positive correlations with all three CCPs, while the Confrontational style produced negative correlations. For the middle range of RSSQ scales there was less uniformity; Eliciting had a positive association with guiding sessions in a productive manner, while Sustain Talk was negatively linked with Empathy.

As reviewed earlier, several previous tools measuring MI competence have shown a sensitivity to the impacts of MI training with forensic practitioners (MITI, VASE-R, ORQ, WRQ, MIKAT). In the final hypothesis, we predicted that RSSQ scales would also show patterns of improvement following a training program, as well as differences among practitioners with MI training experiences compared to those with no previous MI exposure. Following a CBT training program, POs showed significantly less Confrontation and greater Eliciting, and small improvements in the Change Talk style. Correspondingly, in the original sample of forensic practitioners, those who came in with prior MI training scored better on most scales than those with no MI background—scoring significantly lower on the Confrontational style and significantly higher on the Eliciting and Change Talk styles. In both samples, the Sustain Talk scale was relatively unaffected or unrelated to training experiences. These findings are likely an underestimate of the RSSQ’s ability to assess the impacts of MI training because the PO training curriculum was mainly CBT focused and contained only elements of MI, while the marker of previous MI training from the original sample was imprecise and did not delve into the extent and recency of practitioners MI training histories.

### Implications

The major difference between the RSSQ and other practitioner completed tools (e.g., VASE-R, HRQ, ORQ, WRQ) is that respondents consider an assortment of dissimilar responses to clients and identify the degree to which each response is consistent with their own style. The preliminary validation of the RSSQ suggests that such an approach may be a reasonable alternative to open-ended formats that require coding and scoring. In using this approach, additional case scenarios could be developed and tailored to forensic practitioners working in specialized areas such as intimate partner and domestic violence, sex offending, and for those working with adolescents or justice-involved women. Using the same scale construction methodology, assessment tools could also be created for other environments and around specific mental health disorders. Instruments created in this manner would be most appropriate for screening purposes and would not be a replacement for more complex practitioner observation tools such as the MITI and MISC that better capture nuances of MI practice. A practical distinction is that tools such as the RSSQ can be most helpful for gauging a group of practitioners before training initiatives, while practitioner observation tools can be more useful for improving proficiency through individual feedback and ongoing coaching.

The scales (i.e., styles) of the RSSQ emerged along a continuum of MI-inconsistent to MI-consistent. The Confrontational style reflects the most extreme form of non-adherence to MI principles followed by the Sustain Talk style. The Change Talk style reflects the greatest alignment with MI strategy followed by the Eliciting style. It is not surprising that some of the strongest findings occurred on the scales representing the more extreme practitioner response styles.

The MI inconsistent-consistent continuum that emerged across the three studies suggest a tale of two different PO outlooks on the nature of the profession. In particular, POs with a Confrontational style seemed to possess a toxic constellation of characteristics such as an authoritarian view of the job, extrinsic motivation, poor utilization of agency resources, lack of empathy, poor communication skills, and a non-collaborative stance with clients. As described by Chudzik and Aschieri (2013), these POs seem to possess a detached indifference to the change process sometimes observed among forensic practitioners.

As noted by Magill et al. (2018), it may be particularly important to identify practitioner behaviors that are inconsistent with MI principles. The Confrontational scale may have the potential to detect practitioners who may be causing harm and who need additional observation and training. We speculate that practitioners with a strong Confrontational style may pose management challenges to agencies and programs that value improving client behavior and reducing recidivism. Future research might explore the relationship between RSSQ styles and various metrics of practitioner job performance (e.g., supervisor evaluations, official client complaints, and disciplinary actions). On a more positive note, based on the results in Study 3, it appears that a Confrontational style may be somewhat modifiable through training experiences.

Alternatively, POs with a Change Talk style viewed themselves as playing an active role in supporting their clients in behavior change. They were also internally motivated, likely to utilize and see value in agency resources, possessed proficient communication skills, displayed empathy, and tended to engage clients in a collaborative manner. These are qualities that criminal justice agencies and programs seek to foster in their staff. The RSSQ may be useful for identifying practitioners with higher levels of skill, expertise, aptitude, and talent.

MI provides a platform of clinical skills for a diverse—in terms of education, attitudes, training, previous employment, life experiences—population of practitioners who often enter the field with a narrow range of competencies for interacting with justice-involved clients (Tafrate et al., 2019). Since MI skills are foundational, they are frequently used in combination with other evidence-based practices (Miller and Rollnick, 2023, pp. 302–303). The RSSQ may have applicability as a screener for basic skills in models—other than MI—that are commonly used in forensic settings such as CBT (Mitchell et al., 2023), Risk-Need-Responsivity (Bonta, 2023), and CCPs (Lowenkamp et al., 2012). In fact, in Study 3 with a small sample of POs, the majority of RSSQ scales showed improvements following a CBT training program.

### Strengths, limitations, and future directions

A strength of these initial validation studies was that they were conducted in criminal justice environments with forensic practitioners. Nonetheless, several limitations exist. First, the RSSQ was not directly compared with more rigorous practitioner observation tools such as the MISC (Houck et al., 2010) or MITI (Moyers et al., 2014, 2016), or a more established practitioner completed tool such as the ORQ (Walters et al., 2008). Future research should further explore the convergent validity of the RSSQ with previously validated instruments that measure MI fidelity and skill. Another limitation concerns the ability of RSSQ scales to measure the effects of training. Although the majority RSSQ scales appear sensitive to MI training experiences, future studies should use the RSSQ to assess practitioners participating in an MI specific training curriculum (as opposed to a CBT-based program). Finally, practitioner scores on the RSSQ have not yet been linked to important criminal justice *client* outcomes such as program attrition and rearrest. As noted in the review by Madson et al. (2019), a connection between practitioner skills and client outcomes is a weakness across the MI assessment literature in general.

## Conclusion

By its nature, forensic work encompasses a spectrum of functions and responsibilities as practitioners balance the interests of the courts, clients, and victims (Chudzik and Aschieri, 2013). Across criminal justice settings, there exists a tension between the monitoring and sanctioning aspects of the job and the rehabilitative and helpful components. The degree to which practitioners emphasize these perspectives, as well as their ability to communicate skillfully with clients, characterizes their interactions. As a screening tool to assess an MI practice orientation, the RSSQ shows promise as an efficient and useful indicator of forensic practitioner’s response styles.

## Data availability statement

The datasets presented in this article are not readily available because restrictions apply to the availability of the data for this study. Availability is subject to approval from the State of Connecticut Judicial Branch Court Support Services Division. Requests to access the datasets should be directed to RT, tafrater@ccsu.edu.

## Ethics statement

The studies involving humans were approved by Central Connecticut State University. The studies were conducted in accordance with the local legislation and institutional requirements. The participants provided their written informed consent to participate in this study.

## Author contributions

RT: Writing – original draft. DM: Writing – original draft. SC: Writing – original draft. TH: Writing – original draft.

## Footnotes

^1^For an exception see Walters et al. (2010) noted below. In this study, MITI coded work samples were obtained from role-plays between probation officers and actors playing different client scenarios.

^2^A caution is warranted about simply asking people how skillful they are in using MI in their work. Practitioners often overestimate their own proficiency in practice (Miller and Rollnick, 2023, pp. 267–284) and practitioner self-report has not been shown to be a valid indicator of objectively measured skill (Miller and Mount, 2001; Wain et al., 2015).

^3^For readers interested in the original item pool, please contact the first author.
