# Supplementary material for: Measuring a motivational interviewing practice orientation in criminal justice practitioners: initial validation of the response style screening questionnaire
Source: Front Psychol. 2023 Dec 6;14:1308086. doi: 10.3389/fpsyg.2023.1308086 (PMC10746862; doi:10.3389/fpsyg.2023.1308086)
Supplement: Supplementary file 1 [file Data_Sheet_1.pdf]

Response Style Screening Questionnaire (RSSQ): Instructions and Scoring Guidelines

Instructions

Practitioners have different styles of responding to some of the challenging statements made by justice-involved clients. In the next few pages, five scenarios are presented that consist of statements a justice-involved client might say during a supervision or case management session. For each scenario, imagine you are in the beginning phase of working with this person, and that you are trying to set the expectations and tone for future meetings. Consider each statement as if the client was actually talking to you.

A number of potential responses that a practitioner might make follow each client statement. Rate how likely it would be **for you** to make such a response to a client in this situation by circling the number that corresponds to the choices outlined in the box below. There are no right or wrong answers. Just evaluate how close **each** practitioner response is to your own natural style. If you have not encountered a situation like some of the scenarios presented, then simply imagine how you would typically respond.

I would **never** respond this way. This is not at all consistent with my natural style of reacting to a client in this type of situation.

I would **rarely** respond this way. This response is not typical of my style for interacting with a client in this type of situation.

I might **sometimes** respond this way. I could see myself saying something like this with certain people. This response would be somewhat consistent with my style of responding.

I would **frequently** respond this way. This is very close to my natural style of reacting to a client in this situation.

<u>Scenario #1:</u> A 22-year-old man tells you: *"I want to stay clean and sober, but I can't afford to get my own place yet; so, I have to live with my brother who drinks all the time."*

<u>Practitioner response (a):</u> *"The bottom line is if you start using again, you're going to get violated. I don't know how you're going to handle this, but you need to remember that your brother isn't going to be doing your time for you."*

I would likely respond this way… 1-----------2-------------3------------4
Never Rarely Sometimes Frequently

<u>Practitioner response (b):</u> *"Part of you really wants to stay clean, but the situation with your brother makes that difficult."*

I would likely respond this way… 1-----------2-------------3------------4
Never Rarely Sometimes Frequently

<u>Practitioner response (c):</u> *"It sounds like if you start using again, it will definitely be because of your brother's negative influence."*

I would likely respond this way… 1-----------2-------------3------------4
Never Rarely Sometimes Frequently

<u>Scenario #2:</u> A 39-year-old man tells you: *"I'm not a criminal, I'm an addict. You don't understand what it is like. It's not like I can just stop. I know you just want to keep taking urines on me so I go to jail. You need to help me.*"

<u>Practitioner response (a):</u> *"It seems like you are really looking for some help at this point."*

I would likely respond this way… 1-----------2-------------3------------4
Never Rarely Sometimes Frequently

<u>Practitioner response (b):</u> *"Sounds like you're an addict, and it would be almost impossible for you to change."*

I would likely respond this way… 1-----------2-------------3------------4
Never Rarely Sometimes Frequently

<u>Practitioner response (c):</u> *"When you choose to break the law there are consequences. Drug use is a choice. If you end up in jail it won't be because of me. It will be because you made the wrong choices."*

I would likely respond this way… 1-----------2-------------3------------4
Never Rarely Sometimes Frequently

<u>Scenario #3:</u> A 44-year-old man tells you: *"You're damn right I'm angry. The system is unfair to men. She should be here and not me. She starts all the arguing and doesn't know when to stop. The sad thing is that my kids are affected by all this fighting and drama."*

<u>Practitioner response (a):</u> *"What kind of things do you argue about?"*

I would likely respond this way… 1-----------2-------------3------------4
Never Rarely Sometimes Frequently

<u>Practitioner response (b):</u> *"Sounds like you have some real concerns over how all this arguing is affecting your kids."*

I would likely respond this way… 1-----------2-------------3------------4
Never Rarely Sometimes Frequently

<u>Practitioner response (c):</u> "*Sounds like she is to blame for most of what has happened."*

I would likely respond this way… 1-----------2-------------3------------4
Never Rarely Sometimes Frequently

<u>Practitioner response (d):</u> *"However all this came about, you need to make the best of it now. Just do what the judge required, and you can put all this behind you."*

I would likely respond this way… 1-----------2-------------3------------4
Never Rarely Sometimes Frequently

<u>Practitioner response (e):</u> *"First of all, watch your tone and don't swear in my office. You're not talking to your girlfriend now; you're talking to me."*

I would likely respond this way… 1-----------2-------------3------------4
Never Rarely Sometimes Frequently

<u>Scenario #4:</u> A 24-year-old woman tells you: *"I know I sometimes make poor decisions. I've had a lot of things happen to me; there's things that bother me. I get depressed. I get emotional just talking about it. But I don't think I'm crazy. I'm not sure about counseling. I don't see how talking about my problems will help anything. It will probably make me feel worse."*

<u>Practitioner response (a):</u> *"There's no way of knowing how you'll feel until you've tried it. In any event, counseling is required as part of your probation. You won't feel very good if you end up back in court on a violation."*

I would likely respond this way… 1-----------2-------------3------------4
Never Rarely Sometimes Frequently

<u>Practitioner response (b):</u> *"It seems like you would not be able to handle going to counseling."*

I would likely respond this way… 1-----------2-------------3------------4
Never Rarely Sometimes Frequently

<u>Practitioner response (c):</u> *"Tell me more about your history of depression."*

I would likely respond this way… 1-----------2-------------3------------4
Never Rarely Sometimes Frequently

<u>Scenario #5:</u> A 52-year-old man tells you: *"I don't really think I should be here. I'm not an abuser. It was just an argument that got out of hand."*

<u>Practitioner response (a):</u> *"Tell me more about what led up to the argument."*

I would likely respond this way… 1------------2--------------3-------------4
Never Rarely Sometimes Frequently

<u>Practitioner response (b):</u> *"I'll bet if we took a poll of everyone else sitting in the waiting room, none of them think they should be here either. This program isn't voluntary. It's a court order."*

I would likely respond this way… 1------------2--------------3-------------4
Never Rarely Sometimes Frequently

<u>Practitioner response (c):</u> *"How long have you been in this relationship?"*

I would likely respond this way… 1------------2--------------3-------------4
Never Rarely Sometimes Frequently

<u>Practitioner response (d):</u> "*You sound concerned and embarrassed that this argument got so out of hand."*

I would likely respond this way… 1------------2--------------3-------------4
Never Rarely Sometimes Frequently

Scoring and Interpreting the RSSQ

Scoring is based on the type of verbalizations that each practitioner response is likely to produce from the client. Practitioner responses are grouped across the four response styles outlined below. Score each response style by calculating a total for the items and taking the average. Scores range from 1 to 4, with higher scores indicating greater endorsement of the response style being measured. For example, higher scores on the Confrontational and Sustain Talk styles indicate greater MI-nonadherence, while higher scores for Eliciting and Change Talk styles reflect more alignment with an MI-practice orientation. Preliminary benchmarks are based on 1,187 forensic practitioners who completed the RSSQ in the initial validation studies.

Confrontational style responses are likely to produce discord, increase client resistance, and have the potential to be harmful. **Items: 1a, 2c, 3d, 3e, 4a, 5b**
Benchmark: (M = 1.77, SD = 0.60; 25th percentile = 1.33, 50th percentile = 1.67, 75th percentile = 2.17)

Sustain Talk style responses are likely to elicit sustain talk and may inadvertently reinforce helplessness or justifications for not changing. **Items: 1c, 2b, 3c, 4b**
Benchmark: (M = 1.50, SD = 0.51; 25th percentile = 1.00, 50th percentile = 1.25, 75th percentile = 1.75)

Eliciting style responses are likely to increase client verbalizations and elicit new information. **Items: 3a, 4c, 5a, 5c**
Benchmark: (M = 3.33, SD = 0.58; 25th percentile = 3.00, 50th percentile = 3.50. 75th percentile = 3.75)

Change Talk style responses focus on the parts of client statements most related to change and are likely to evoke change talk and reinforce motivation for change. **Items: 1b, 2a, 3b, 5d**
Benchmark: (M = 3.43, SD = 0.50; 25th percentile = 3.00, 50th percentile = 3.50, 75th percentile = 3.75)
